# Analysis of the safety and efficacy of the self-pulling and latter transected technique in modified overlap anastomosis in total laparoscopic total gastrectomy

**DOI:** 10.3389/fonc.2024.1334141

**Published:** 2024-05-24

**Authors:** Jintian Wang, Jing Xiong, Pengcheng Wang, Jianan Lin, Wenjin Zhong, Wengui Kang, Chuying Wu, Junxing Chen, Huida Zheng, Kai Ye

**Affiliations:** ^1^ Department of Gastrointestinal Surgery, The Second Affiliated Hospital of Fujian Medical University, Quanzhou, China; ^2^ Department of Nursing, The Second Affiliated Hospital of Fujian Medical University, Quanzhou, China

**Keywords:** self-pulling and latter transected, total laparoscopic total gastrectomy, overlap anastomosis, digestive tract reconstruction, linear stapler

## Abstract

**Background:**

Laparoscopic total gastrectomy plus lymph node dissection is an effective treatment method for patients with gastric cancer. With the development and popularization of laparoscopic techniques in recent years, surgeons have become more skilled in laparoscopic techniques. Totally laparoscopic total gastrectomy (TLTG) has been developed; however, digestive tract reconstruction remains difficult, especially with anastomosis of the esophagus and jejunum. Using the self-pulling and latter transection (SPLT) method combined with a linear stapler has effectively solved the problem of narrow space in esophagojejunostomy. Here, we examined the safety and effectiveness of the SPLT technique in TLTG compared with SPLT with traditional esophagojejunostomy overlap anastomosis.

**Methods:**

We retrospectively analyzed all patients with gastric cancer admitted to the Department of Gastrointestinal Surgery of the Second Affiliated Hospital of Fujian Medical University from September 2020 to September 2023. In total, 158 patients met the inclusion criteria and were included. Patients were grouped according to whether the lower esophagus was transected after self-pulling. Patient demographics, tumor characteristics, surgical conditions, and postoperative results between the two groups were statistically analyzed.

**Results:**

A total of 158 patients were included in the study. All patients underwent TLTG and completed intracavitary anastomosis. There were 70 cases (44%) in the SPLT-Overlap group and 88 cases (56%) in the traditional overlap group. There was no significant difference in demographic and oncological characteristics between the two groups. The operation time (P = 0.002) and esophageal jejunum anastomosis time (P<0.001) were significantly shorter in the SPLT-Overlap group compared with the traditional overlap group. The intraoperative blood loss of the SPLT-Overlap group was 80.29 ± 36.36 ml, and the intraoperative blood loss of the traditional overlap group was 101.40 ± 46.68 ml. The difference was statistically significant (P=0.003). The SPLT-Overlap group also achieved a higher upper cutting edge (P =0.03). There was no significant difference between the two groups in terms of the incision size, postoperative hospital stay, time to first flatus, time to first liquid intake, drainage tube removal time, and esophagojejunal anastomotic diameter. There were 15 and 19 cases of short-term postoperative complications in the SPLT-Overlap and traditional Overlap groups, respectively. All patients received R0 resection, and no secondary surgery or death occurred.

**Conclusion:**

We applied SPLT to overlap anastomosis. Short-term, SPLT has good safety and feasibility in TLTG. It can effectively shorten the time of digestive tract reconstruction, simplify the reconstruction procedure, and make the digestive tract reconstruction simple and fast; at the same time, a safe cutting edge can be obtained.

## 1 Introduction

In 1994, Professor Kitano and his team first reported laparoscopic radical gastrectomy for gastric cancer (1). With the rapid development of laparoscopic instruments and techniques, the application of laparoscopic techniques in radical gastrectomy for gastric cancer has become more widespread. Studies have shown that the 5-year survival rate of patients with locally advanced gastric cancer treated by laparoscopy is not inferior to that of open surgery (2).

Digestive tract reconstruction is one of the difficulties and hotspots in laparoscopic radical gastrectomy for gastric cancer. Anastomosis of the esophagus and jejunum determines the success or failure of the operation, and is closely related to the occurrence of postoperative anastomotic complications (3, 4). The traditional laparoscopic-assisted anastomosis technique needs to be conducted through the median abdominal incision, while total laparoscopic esophagojejunostomy overcomes this disadvantage. The main surgical methods include reverse puncture (5), Orvil, functional end-to-end esophagojejunostomy (FETE), Overlap (6), and π anastomosis. At present, the Overlap method is widely used in China. Its main advantage is that it can be completely carried out under laparoscopy. The esophagus and jejunum are in iso-peristaltic anastomosis, and the size of the anastomosis is not limited by the diameter of the esophagus and jejunum, thus avoiding the difficulty of placing the anchor seat in the traditional laparoscopic assisted anastomosis technology.

In 2017, Professor Hao innovatively applied SPLT technology to totally laparoscopic esophagojejunostomy for the first time in China (7). Because the Overlap method is a side-to-side anastomosis, a sufficient length of the esophageal stump needs to be freed to facilitate the placement of the stapler. The SPLT technique uses the traction of the lower esophagus to provide tension, avoiding the high retraction of the stump after the esophagus is severed, and it is difficult to close the common opening. At the same time, it can ensure the integrity of the cutting edge, and has a good application prospect.

## 2 Patients and methods

### 2.1 Patients

The inclusion criteria were as follows: preoperative examination confirmed Siewert type II or Siewert type III esophagogastric junction cancer; and preoperative upper abdominal enhanced computed tomography (CT) suggested T1–3 and no distant metastasis (M0). The exclusion criteria were as follows: Siewert type I; patients with severe heart, brain, liver, lung, kidney, and other dysfunction who could not tolerate surgery; and malignant tumors in other areas. In the included patients, complete laparoscopic total gastrectomy and D2 lymph node dissection were performed, and side-to-side esophagojejunostomy was performed. Patients were informed of the advantages and disadvantages of the two procedures before surgery, and the surgical procedure was selected by signing an informed consent form.

All operations were performed by the same team with more than 10 years of surgical experience. Postoperative routine anti-infection, nutritional support, analgesia, fluid infusion, and other treatments were performed. According to the clinical guidelines of the Chinese Society of Clinical Oncology (CSCO) (8) for gastric cancer, patients with cT3–4aN_+_M0 (Stage cII–cIII) were treated with the SOX regimen for neoadjuvant therapy. To assess the efficacy, we evaluated tumor marker detection, electronic gastroscope, enhanced CT, symptoms, and signs. According to the patient’s state, the timing of surgery was generally 3–6 weeks after the end of neoadjuvant therapy.

The study was reviewed and approved by the Ethics Committee of the Second Affiliated Hospital of Fujian Medical University. According to national legislation and institutional requirements, this study does not require written informed consent to participate.

### 2.2 Common surgical procedures

**Figure 1:**
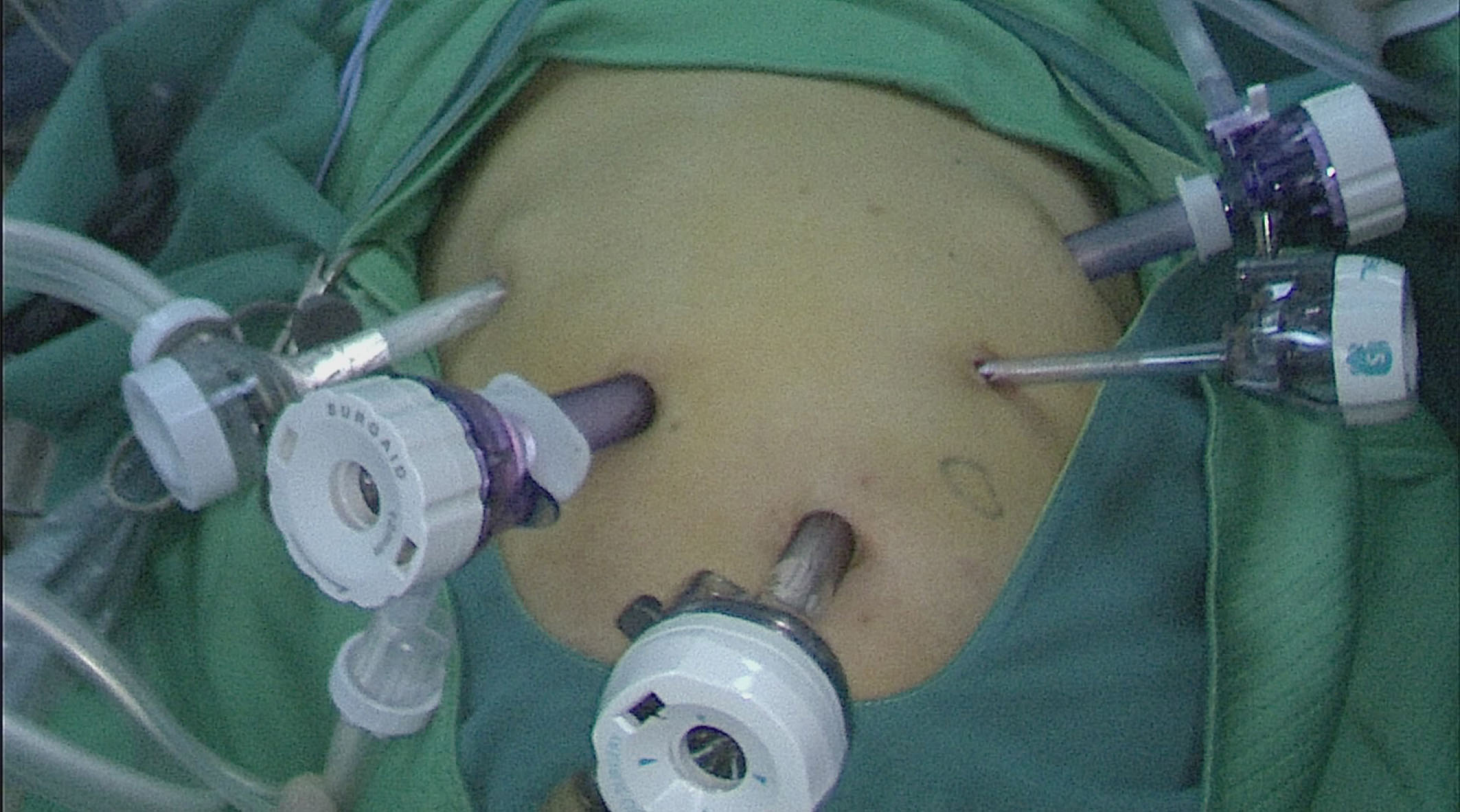
The distribution of Troca holes in TLTG. The specimens were taken out through the median incision of the upper abdomen. TLTG, totally laparoscopic total gastrectomy.

The patients were placed in the supine split leg position. Once under general anesthesia, the skin of the surgical field was routinely disinfected and covered with sterile surgical towels. The surgeon stood on the left side of the patient, the assistant stood on the right side, and the cameraman stood between the patient’s legs. The operation was performed using the five-hole method. First, a 12 mm Trocar was placed under the umbilicus as an observation hole. After establishing the pneumoperitoneum, the abdominal cavity was explored. After confirming that there was no metastasis, under its guidance, a 12 mm Trocar and a 5 mm Trocar were placed at 2 cm below the costal margin of the left anterior axillary line and 2 cm above the umbilicus of the left midline of the clavicle, respectively, and a 5 mm Trocar and 12 mm Trocar were placed at the corresponding positions on the right side (**Figure 1**).

The liver was routinely suspended with a purse-string needle to fully expose the surgical field and better complete the esophagojejunostomy. Laparoscopic total gastrectomy and D2 lymph node dissection were conducted in line with standard procedures (9, 10). After D2 lymph node dissection, vascular disconnection, and whole stomach dissociation, the front of the diaphragm esophageal hiatus and the left diaphragm foot were opened, the lower end of the esophagus was fully dissociated, and the duodenum was cut off with a linear cutting closure device; then, the anastomosis procedure was performed.

### 2.3 SPLT-overlap procedures

**Figure 2:**
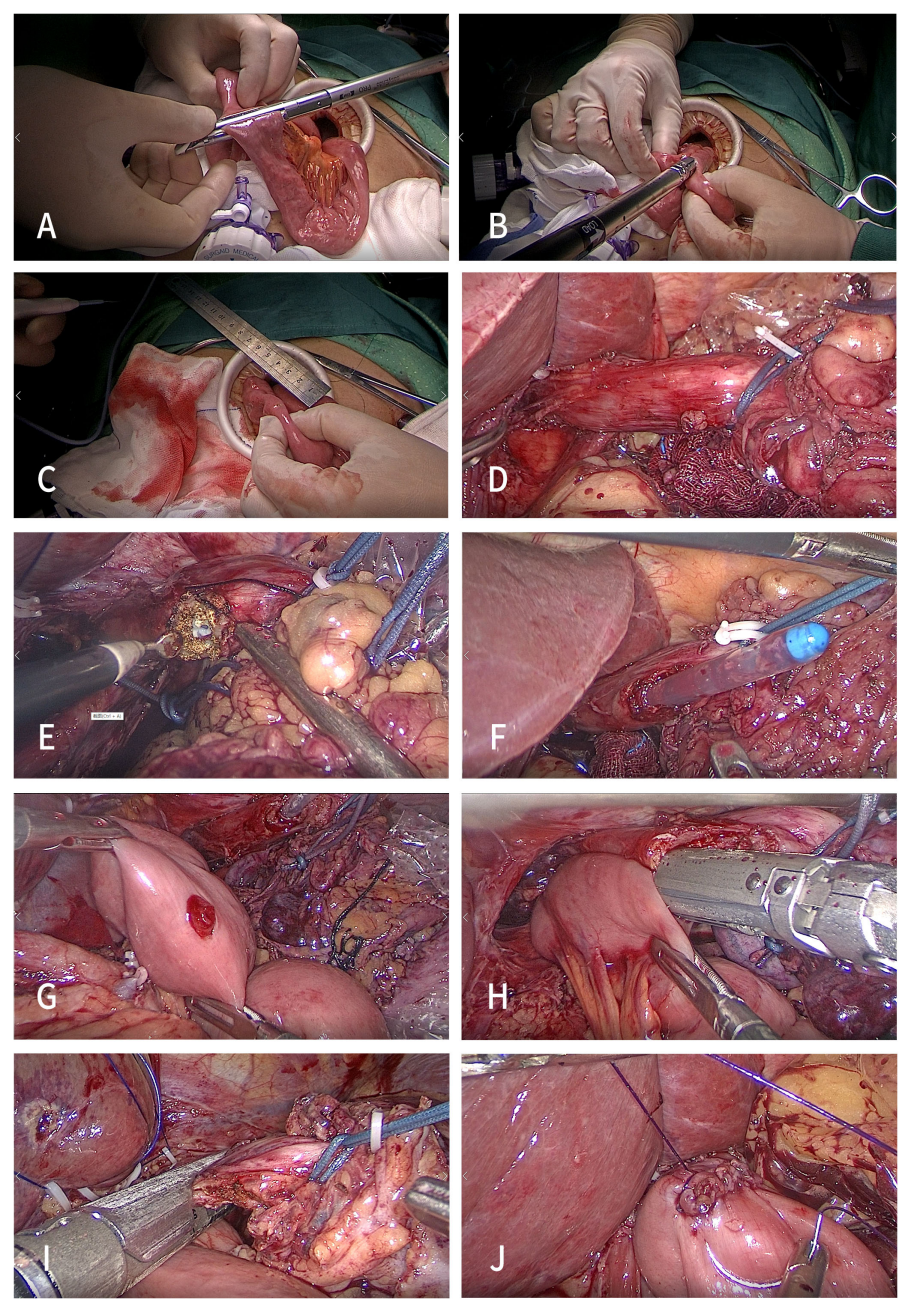
Procedures of the SPLT-Overlap group. **(A)** Disconnect the jejunum and its mesentery. **(B)** Jejunal side-to-side anastomosis. **(C)** Puncture the distal jejunum at 5 cm. **(D)** Ligate the esophagus. **(E)** Expose the esophageal mucosa. **(F)** Gastric tube guided anastomosis. **(G)** Expose the common opening of the jejunum. **(H)** Side-to-side esophagojejunostomy. **(I)** Disconnect the esophagus. **(J)** Close the common opening. TLTG, totally laparoscopic total gastrectomy; SPLT-Overlap, self-pulling and latter transection overlap anastomosis.

The esophagus was not cut off temporarily. The whole stomach specimen after the duodenum was cut off was placed into the specimen bag. The pneumoperitoneum was closed, and an auxiliary small incision of approximately 5 cm was made in the middle of the upper abdomen below the xiphoid process. The jejunum and its mesentery were disconnected at a distance of approximately 20–25 cm from the Treitz ligament (**Figure 2A**). Jejunal side-to-side anastomosis of the input loop and the output loop was performed at a distance of approximately 40 cm from the esophagojejunal anastomosis (**Figure 2B**). The intestinal wall of the opposite side of the mesentery at the distal jejunum approximately 5 cm away from the broken end was punctured for use as a common opening for esophagojejunostomy (**Figure 2C**).

The pneumoperitoneum was re-established, and the assistant placed an approximately 15 cm long sterile rope through the right 12 mm Trocar hole, and ligated the esophagus on the cephalic side of the upper edge of the tumor (Siewert type II) or the esophagogastric junction (Siewert type III) (**Figure 2D**). Then, in their right hand, the assistant used a laparoscopic needle holder to pull the esophagus to the ventral caudal side. The surgeon performed esophageal hiatus and lower mediastinal lymph node dissection under the condition of esophageal self-pulling, while freeing the lower esophagus. Then, the right posterior wall of the esophagus at the proximal end of the ligation line was cut with a power shovel parallel to the ligation line from the back to the ventral side to expose the esophageal mucosa (**Figure 2E**), and the anastomosis channel was confirmed under the guidance of the gastric tube (**Figure 2F**).

The distal jejunum was pulled close to the esophageal ligation line through the posterior direction of the transverse colon. Using their left hand, the assistant used the gastric forceps to pull the jejunal stump to the head side, and the surgeon used their left hand to use the intestinal forceps to pull the tail side. The tension fully exposed the jejunum opening in the field of vision (**Figure 2G**). Then, the surgeon placed a 45 mm linear stapler from the upper left 12 mm Troca, and placed the stapled surface of the linear stapler into the jejunal opening. The non-studded surface was placed into the esophageal opening under the guidance of the gastric tube. At this time, the assistant used their right hand to use the laparoscopic needle holder to pull the esophagus to the ventral tail to form a self-pulling state again and complete the side-to-side anastomosis of the esophagus and jejunum (**Figure 2H**).

After anastomosis, the perforation, false passage, and bleeding were carefully examined. Then, the assistant placed a 45 mm linear stapler from the 12 mm Troca hole on the right side, and used their left hand to pull the sterile rope downward, forming a self-pulling state for the third time to complete the disconnection of the esophagus (**Figure 2I**). The common opening was manually closed using a barbed wire under laparoscopy (**Figure 2J**). Conventional barbed wire embedding duodenal stump. The specimens were taken out through the abdominal median incision.

### 2.4 Traditional overlap procedures

After the dissociation was completed, the esophagus was directly disconnected with a 45mm linear stapler, and then the pneumoperitoneum was closed. The specimen was taken out through the median abdominal incision and the pneumoperitoneum was re-established. The gastric tube was used to hold the esophageal stump, and the surgeon relied on the pulling force of the gastric forceps to pull the stump to avoid its high retraction. Then, the right posterior wall of the esophagus was cut with a power shovel parallel to the stump from the back to the ventral side to expose the esophageal mucosa. Under the guidance of the gastric tube, the anastomosis channel was confirmed and the anastomosis was completed. The subsequent surgical procedure was consistent with the procedure SPLT. A drainage tube was placed around the anastomosis and the splenic hilum, and the abdominal incision was closed to end the operation.

### 2.5 Postoperative management

**Figure 3:**
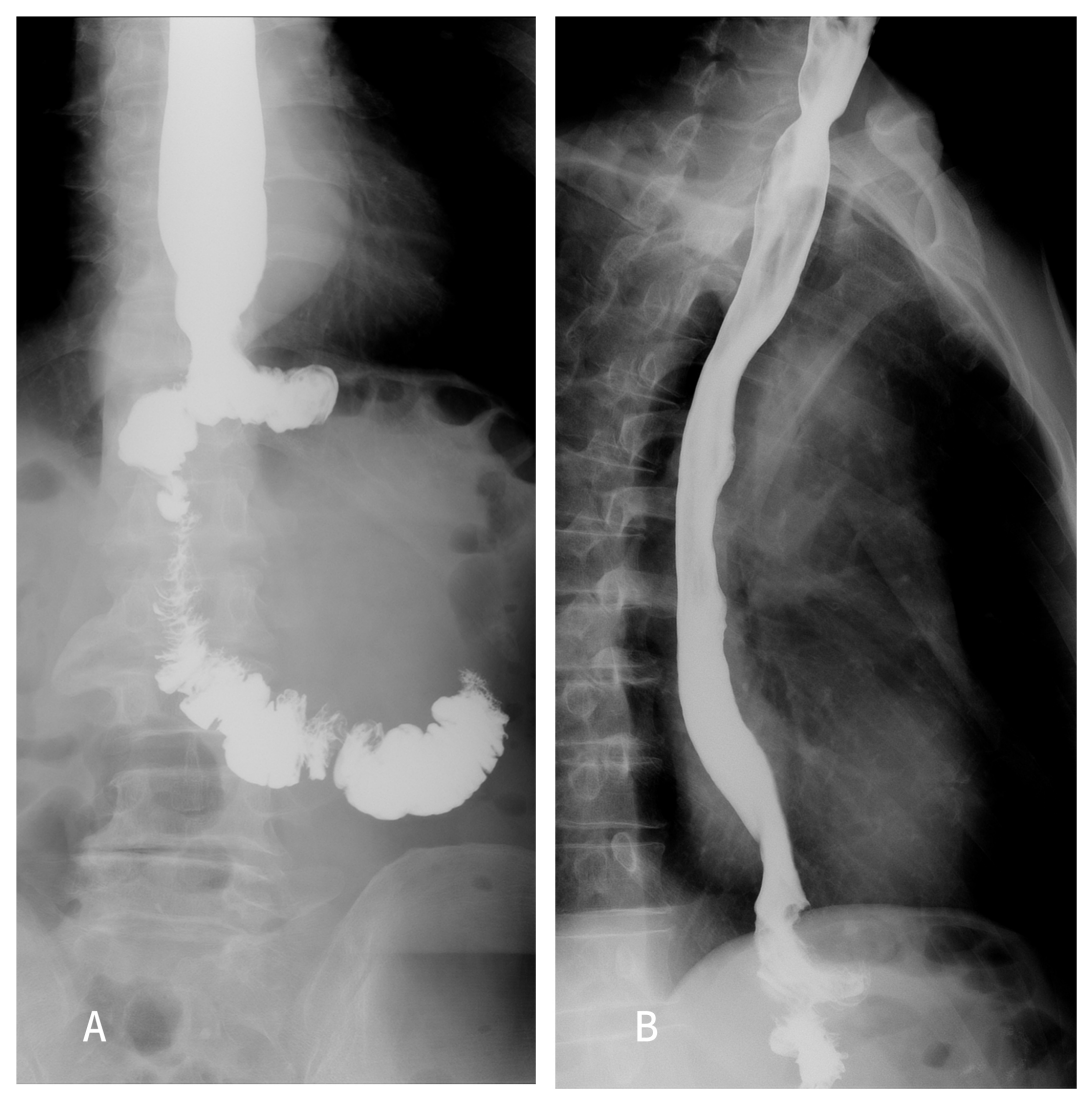
Upper gastrointestinal radiography was performed 5 days after the operation in the SPLT-Overlap group. **(A)** Coronal position. **(B)** Sagittal position. SPLT-Overlap, self-pulling and latter transection overlap anastomosis.

All patients received standardized postoperative management. Broad-spectrum antibiotics were used for 48 h after the operation, and octreotide was routinely used until open diet. Blood routine and procalcitonin were continuously monitored 3 days after the operation. If the patient was in good condition, upper gastrointestinal water-soluble angiography (**Figure 3**) was usually performed on the third day after surgery to exclude anastomotic leakage. An open liquid diet after smooth exhaust and defecation. After excluding the recent postoperative complications, the abdominal drainage tube was removed. After 1 day of observation, if the patient had no discomfort, they could be discharged.

### 2.6 Data collection

The demographic data of the two groups were collected, including age, sex, body mass index (BMI), preoperative albumin, preoperative hemoglobin, American Society of Anesthesiologists (ASA) score, smoking and drinking history, abdominal surgery history, preoperative tumor with obstruction, and bleeding. Intraoperative data were collected, such as the operation time, esophagojejunostomy time, intraoperative blood loss, length of the upper cutting edge, and incision size. Postoperative data included the postoperative hospital stay, time to first flatus, time to first liquid intake, drainage tube removal time, and esophagojejunal anastomotic diameter. Postoperative complications included anastomotic leakage, anastomotic bleeding, anastomotic stenosis, duodenal stump leakage, pancreatic fistula, postoperative bleeding, internal abdominal hernia, postoperative ileus, chyle leakage, wound infection, pulmonary infection, pleural effusion, abdominal abscess or effusion, abdominal infection, secondary surgery, and perioperative death. Postoperative complications were compared using the Clavien–Dindo classification system (11). The system divides postoperative complications into five grades (**Table 1**).

**Table 1:** Clavien–Dindo complication grading system.

Grade	Definition
Grade I	Any deviation from the normal postoperative course without the need for pharmacological treatment or surgical, endoscopic, and radiological interventions The allowed therapeutic regimens are: drugs as antiemetics, antipyretics, analgesics, diuretics, electrolytes, and physiotherapy. This grade also includes wound infections opened at the bedside
Grade II	Requiring pharmacological treatment with drugs other than those allowed for grade I complications Blood transfusions and total parenteral nutrition are also included
Grade III	Requiring surgical, endoscopic or radiological intervention
Grade IIIa	Intervention not under general anesthesia
Grade IIIb	Intervention under general anesthesia
Grade IV	Life-threatening complication (including CNS complications)* requiring IC/ICU management
Grade IVa	Single organ dysfunction (including dialysis)
Grade IVb	Multiorgan dysfunction
Grade V	Death of a patient

*Brain hemorrhage, ischemic stroke, subarrachnoidal bleeding, but excluding transient ischemic attacks.

CNS, central nervous system; IC, intermediate care; ICU, intensive care unit.

### 2.7 Statistical analysis

All statistical analyses were performed using SPSS (version 25.0; IBM Inc., Armonk, NY, USA) and GraphPad Prism software (version 9.0; GraphPad Software Inc., Boston, MA, USA). The measurement data with a normal distribution are expressed as mean ± standard, and the Student’s *t*-test was used for comparison between groups. Continuous variables with a non-normal distribution are shown as M (range), and the Mann–Whitney U test was used for comparison between groups. The count data are expressed as the absolute number or percentage, and the comparison between groups was performed using the Chi-square test or Fisher’s exact probability method. Categorical data were compared using the rank sum test. P<0.05 was considered to indicate a statistically significant difference.

## 3 Results

A total of 158 patients were included in this study. We compared the patient demographics and oncological characteristics of the two groups (**Table 2**). No significant inter-group differences were found in the sex, age, BMI, preoperative albumin and hemoglobin levels, preoperative tumor markers, ASA score, oncological characteristics, comorbidities and tumor complications.

**Table 2:** Patient demographics and oncological characteristics of both groups.

Characteristics	SPLT-Overlap (n=70)	Traditional Overlap (n=88)	P value
Age (years)	69.00 (65.00, 72.00)	66.50 (60.25, 71.75)	0.104
Sex (male/female)	15/55	25/63	0.316
BMI (kg/m2)	22.47 ± 3.46	22.41 ± 3.40	0.923
Preoperative albumin (g/L)	37.05 (33.50, 42.68)	37.95 (33.43, 43.38)	0.733
Preoperative hemoglobin (g/L)	110.00 (99.00, 131.25)	107.00 (97.25, 137.50)	0.914
Preoperative CEA (ng/ml)	3.72 (2.07, 5.85)	3.35 (1.63, 6.31)	0.554
Preoperative CA199 (u/ml)	16.17 (11.96, 19.04)	14.70 (12.00, 17.32)	0.192
ASA score (1/2/3/4/5/6)	0/59/11/0/0/0	2/72/13/1/0/0	0.688^a^
Tumor maximum diameter (cm)	4.50 (3.00, 7.00)	4.50 (3.00, 8.23)	0.639
Siewert type Type II Type III	31 39	38 50	0.889
Neoadjuvant chemotherapy	12	14	0.835
neurovascular invasion	44	55	0.963
T stage (1/2/3/4)	29/10/22/9	37/12/28/11	0.999
N stage (0/1/2/3)	44/9/9/8	55/13/11/9	0.984
TNM stage (I/II/III/IV)	37/15/18/0	47/19/22/0	0.995
Smoking	17	21	0.951
Drinking	10	12	0.907
Abdominal surgery history	4	5	0.993
Stroke history	1	1	0.999^b^
Hypertension	19	21	0.638
CHD	5	6	0.999^b^
T2DM	2	3	0.999^b^
COPD	10	12	0.907
Hepatic sclerosis	2	3	0.999^b^
Renal insufficiency	8	9	0.809
Gout	4	5	0.999^b^
Hyperthyroidism or hypothyroidism	1	2	0.999^b^
Rheumatoid arthritis	1	2	0.999^b^
Preoperative obstruction	13	13	0.522
Preoperative bleeding	14	16	0.772

BMI, body mass index; CEA, carcinoembryonic antigen; CA199, Carbohydrate antigen199; ASA, American Society of Anesthesiologists; CHD, coronary heart disease; T2DM, type 2 diabetes mellitus; COPD, chronic obstructive pulmonary disease; SPLT-Overlap, self-pulling and latter transection overlap anastomosis.

^a^Fisher’s exact probability method.

^b^Continuous modification.

**Figure 4:**
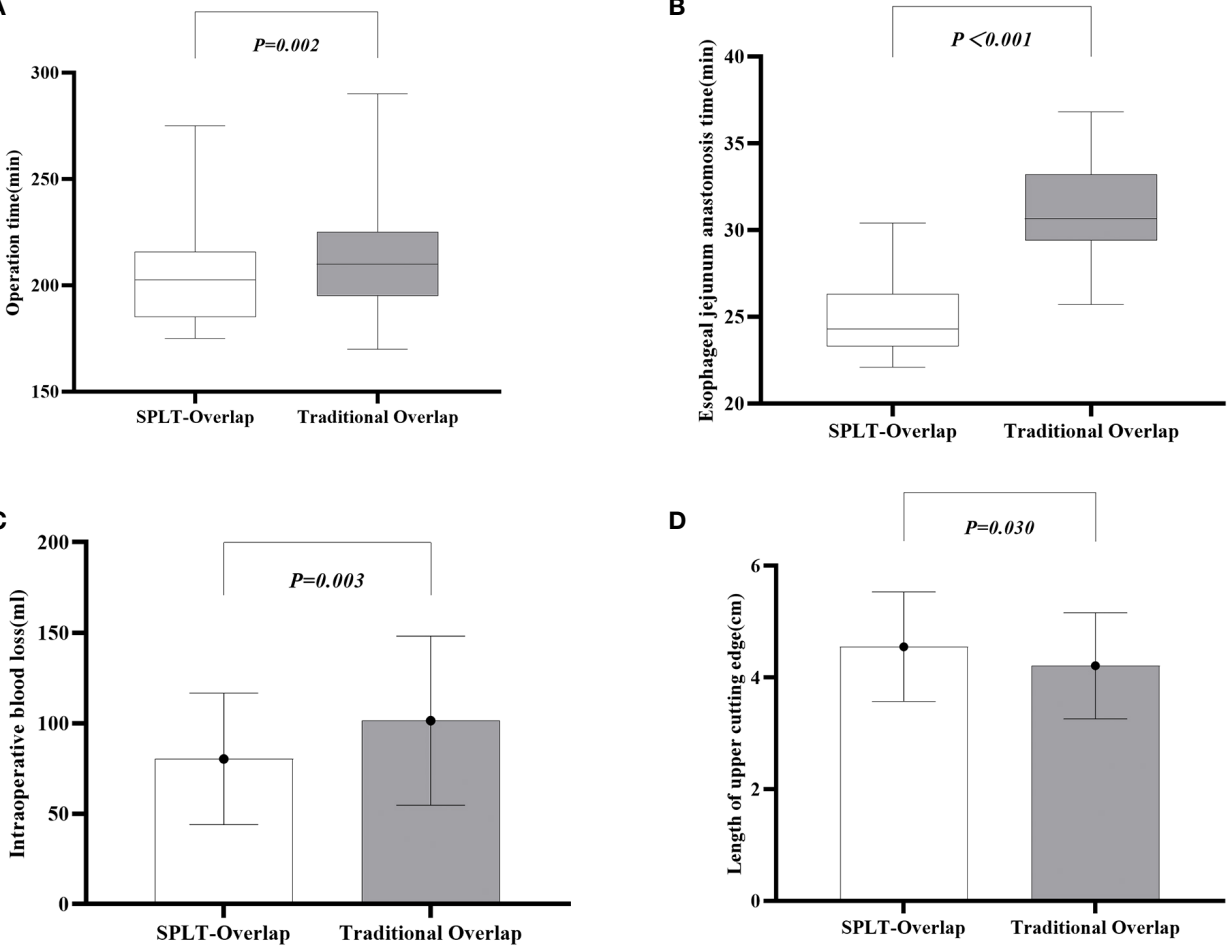
Comparison of surgical conditions between the two groups. **(A)** Operation time. **(B)** Esophageal jejunum anastomosis time. **(C)** Intraoperative blood loss. **(D)** Length of the upper cutting edge. SPLT-Overlap, self-pulling and latter transection overlap anastomosis.

The patient’s surgical conditions and postoperative results are shown in **Table 3**. All patients underwent laparoscopic total gastrectomy and completed intracavitary anastomosis. The SPLT-Overlap group had a significantly shorter operation time (P=0.002) (**Figure 4A**) and esophagojejunostomy time (P<0.001) (**Figure 4B**) compared with the traditional overlap group. The intraoperative blood loss of the SPLT-Overlap group was 80.29 ± 36.36 ml, and that of the traditional overlap group was 101.40 ± 46.68 ml. The difference was statistically significant (P=0.003) (**Figure 4C**). The SPLT-Overlap group also achieved a higher cutting edge (P=0.03) (**Figure 4D**). There was no significant difference between the two groups in terms of the incision size, postoperative hospital stay, time to first flatus, time to first liquid intake, drainage tube removal time, and esophagojejunal anastomotic diameter.

**Table 3:** Comparison of surgical conditions and postoperative results between the two groups.

Characteristics	SPLT-Overlap (n=70)	Traditional Overlap (n=88)	P value
Operation time (min)	202.50 (185.00, 215.75)	210.00 (195.00, 225.00)	0.002
Esophageal jejunum anastomosis time (min)	24.30 (23.30, 36.30)	30.65 (29.40, 33.20)	<0.001
Intraoperative blood loss (ml)	80.29 ± 36.36	101.40 ± 46.68	0.003
Length of upper cutting edge (cm)	4.55 ± 0.98	4.21 ± 0.95	0.030
Incision size (cm)	5.03 ± 0.16	5.05 ± 0.20	0.584
Postoperative hospital stay (days)	10.00 (9.00, 12.00)	10.00 (9.00, 12.00)	0.235
Time to first flatus (days)	3.00 (2.00, 4.00)	3.00 (2.00, 4.00)	0.867
Time to first liquid intake (days)	5.00 (4.00, 7.00)	5.50 (4.00, 7.75)	0.297
Drainage tube removal time (days)	8.00 (7.00, 9.00)	9.00 (8.00, 10.00)	0.336
Esophagojejunal anastomotic diameter (cm)	1.50 (1.50, 1.80)	1.60 (1.50, 1.80)	0.598
Postoperative complications Esophagojejunal anastomotic leakage Grade II Esophagojejunal anastomotic bleeding Esophagojejunal anastomotic stenosis Grade IIIa Jejunal jejunal anastomotic leakage Jejunal jejunal anastomotic bleeding Jejunal jejunal anastomotic stenosis Grade II Duodenal stump fistula Pancreatic fistula Grade II Postoperative bleeding Internal abdominal hernia Postoperative ileus Grade I Grade II Chyle leakage Grade I Grade II Wound infection Pulmonary infection Grade II Pleural effusion Grade II Grade IIIa Abdominal abscess or effusion Grade II Grade IIIa Abdominal infection Grade II Second operation Death	2 2 0 1 1 0 0 1 1 0 1 1 0 0 1 1 0 0 0 0 0 2 2 2 2 0 2 1 1 3 3 0 0	2 2 0 2 2 0 0 0 0 0 1 1 0 0 2 1 1 2 1 1 0 3 3 2 1 1 2 1 1 3 3 0 0	0.980 0.999^a^ 0.999^a^ 0.443^b^ 0.999^b^ 0.999^b^ 0.999^b^ 0.999^a^ 0.769^b^ 0.999^b^ 0.999^a^

SPLT-Overlap, self-pulling and latter transection overlap anastomosis.

^a^Continuous modification.

^b^Fisher’s exact probability method.

There was no significant difference in the incidence of complications between the two groups (P=0.980). Two patients in each group had esophagojejunal anastomotic leakage (all were Clavien–Dindo Grade II), which was improved after a prolonged fasting time, enhanced anti-infection, and abdominal drainage. A total of three patients developed esophagojejunal anastomotic stenosis 1 month after surgery and recovered after endoscopic balloon dilatation (all were Clavien–Dindo Grade IIIa). In the SPLT-Overlap group, there was one case of jejunal jejunal anastomotic stenosis (Clavien–Dindo Grade II), which was considered to be caused by anastomotic inflammatory edema, and the patient recovered after fasting, anti-infection, inhibition of gastrointestinal secretion, and other treatments. There was one case of pancreatic fistula in each group (all were Clavien–Dindo Grade II); the patient improved and was discharged after adequate drainage, infection control, and nutritional support. There were two cases of chylous leakage in the traditional overlap group (one case of Clavien–Dindo Grade I and one case of Grade II), which were cured after fasting, unobstructed drainage, and parenteral nutrition. In addition, patients in both groups experienced postoperative ileus, pulmonary infection, pleural effusion, abdominal abscess or effusion, and abdominal infection, which were also cured after a period of anti-infection, abdominal drainage, and other appropriate treatments. All patients received R0 resection, and no secondary surgery or perioperative death occurred.

Of the 158 patients, 149 were followed up by outpatient or telephone. Of the 70 patients in the SPLT-Overlap group, 66 cases were followed up for 13–39 months, with a median follow-up time of 24 months. Of the 88 patients in the traditional overlap group, 83 cases were followed up for 14–38 months, with a median follow-up time of 23 months. There was one patient with tumor recurrence in the SPLT-Overlap group, and one patient with tumor recurrence and liver metastasis in the traditional overlap group. Both patients are currently in the tumor-bearing survival state. There was no significant difference in tumor recurrence between the two groups (P>0.05). No death occurred.

## 4 Discussion

### 4.1 Development of overlap anastomosis

In the 1960s, traditional gastric cancer surgery relied on manual suture (12) or anastomat (13, 14) for gastrojejunostomy or esophagojejunostomy. With the popularization of minimally invasive technology and the advancement of surgical skills, digestive tract reconstruction technology for gastric cancer has made great progress. With the trend of miniaturization in surgery and refinement of instruments, minimally invasive techniques such as gastrointestinal endoscopic surgery and total endoscopic surgery have gradually become the first choice for the treatment of gastric cancer. The application of the linear stapler makes the anastomosis operation simpler because there is no need for the placement of the nail seat and the purse suture, which reduces the time required for the anastomosis (15) and further shortens the operation time. Studies have shown that the use of a linear stapler can obtain a wider anastomotic diameter and a lower incidence of anastomotic stenosis than a circular stapler (16, 17). A retrospective study in Japan confirmed that it is safe and feasible to use a linear stapler for complete laparoscopic digestive tract reconstruction in patients with gastric cancer, and that it has certain long-term advantages, such as reducing the occurrence of anastomotic stenosis and adhesive intestinal obstruction (18).

Overlap anastomosis is a classical technique using a linear stapler for anastomosis. It was first proposed by Inaba et al. in 2010 (6). Its main advantage is that it improves the corner problem of FETE, adjusts the anastomosis direction of the distal jejunum, reduces the tension of the anastomosis, and is a iso-peristaltic anastomosis, which is more in line with the physiological characteristics of the human body. The disadvantage is that the overlap anastomosis has a distortion of the Roux arm, and the severed esophagus is easily retracted into the mediastinum, thereby increasing the technical difficulty of suturing the common opening. In addition, it is possible to form a submucosal false passage during anastomosis; and under the action of excessive tension, the end of the stapler may break through the jejunum. Therefore, this technique needs to retain a longer lower esophagus to ensure the anastomosis effect, which is generally suitable for tumors invading the lower esophagus within 2 cm. In 2013, Nagai and colleagues improved this technique (19), and the Roux arm distortion and the retraction of the anastomosis were significantly improved. In 2017, Huang proposed an anastomosis technique called the Jejunum-later-cut overlap (20). This technique is based on the traditional overlap anastomosis method, which reduces the degree of distal jejunum dissociation by delaying disconnect the proximal jejunum, and can better control the direction of esophagojejunostomy, thereby reducing the difficulty of surgery. Chen designed an overlap guiding tube (OGT) (21). Through the docking of the OGT and gastric tube, the design increases the controllability of the insertion of the stapler into the esophageal cavity, thereby improving the success rate of the insertion of the stapler into the esophageal cavity at one time and effectively avoiding the formation of esophageal submucosal false passage. These improvements have made the Overlap anastomosis technique widely used in clinical practice, and its effect has also been recognized by many centers (22–24).

### 4.2 Development and application of SPLT technology

In 2017, Chinese scholar Hao reported SPLT for the first time (7). This anastomosis method can obtain a higher esophageal cutting edge, greatly shorten the anastomosis time, reduce intraoperative blood loss, and does not increase the incidence of postoperative complications, which is consistent with the results of this study. Chen applied this technique to laparoscopic distal gastrectomy (25) and Roux-en-Y anastomosis was used for anastomotic reconstruction. A total of 114 patients with gastric cancer were included in the study. They found that the average intraoperative blood loss in the experimental group was significantly lower than that in the control group (P=0.022). In terms of short-term surgical outcomes, they believed that self-pulling was safe and feasible, and had the advantage of simplifying the reconstruction procedure. The Aslan team compared the self-pulling and esophagus latter-cut overlap method anastomosis (SPLCOM) with the overlap method and the Orvil method (26). The results showed that the median anastomosis times of the SPLCOM, overlap, and Orvil groups were 27, 48, and 38 min, respectively (P<0.05). There was no significant difference in the incidence of anastomotic complications between the groups (P=0.299), which was similar to the results of the current study. Since the esophagus is opened when the tumor is not removed, the safety of oncology in the process of disconnection and anastomosis after self-pulling still requires attention, especially for tumors with dentate line invasion. Many scholars have also made various attempts in the reconstruction of self-pulling post-dissection to improve the negative rate of the cutting edge. Qiu et al. suggested that the ligation rope above the tumor can effectively prevent the spread of the tumor (27), and can easily free the area from the lower esophagus to 8–10 cm above the cardia to provide a safe enough distance for anastomosis. Accordingly, they compared the length of the proximal edge of the tumor with esophageal invasion less than 2 cm at the cardia. The data showed no significant difference between the two groups, indicating that SPLT was reliable in terms of tumor safety. For patients with esophageal invasion exceeding 2 cm, they recommended gastroscopy before or during surgery to obtain the exact negative edge. Previous reports have also shown that SPLT can provide a safe and reliable surgical edge (20, 28).

In our study, when performing esophagojejunostomy for the SPLT-Overlap group, we placed the linear stapler from the side of the surgeon to make the direction of the common opening consistent with the stapler, which is more conducive to creating a good anastomotic shape. In the step of closing the common opening, the surgeon will move to the assistant side and use the barbed wire from left to right for a continuous suture. This operation method not only conforms to the habits of the surgeon, but also avoids the tedious knotting steps, which makes the suture easier and more concise. At the same time, it can ensure the exact suture effect and greatly reduce the anastomosis time, thus shortening the operation time. In the SPLT-Overlap group, there was a better operating space and vision, especially for obese patients; this method can be used for more sophisticated operations and reduce tissue trauma. This is also why the blood loss in the SPLT-Overlap group was relatively less. For patients with tumor invasion of the dentate line and neoadjuvant chemotherapy, in order to obtain a reliable margin and ensure R0 resection, we recommend that preoperative ultrasound gastroscopy should be improved to clarify the depth of tumor invasion and regression. Intraoperative gastroscopy can assist us in determining the final margin. When necessary, we believe that intraoperative frozen pathological sections are necessary to obtain an accurate esophageal margin.

### 4.3 Details and advantages of SPLT in modified overlap anastomosis

In this study, SPLT was applied to Overlap anastomosis and appropriately improved. First, we used a sterile rope to pull the lower esophagus. During the anastomosis, two pulling operations were performed to reduce the difficulty of the operation. In addition, this technique avoids the use of laparoscopic instruments to repeatedly clamp the esophageal wall, thereby reducing the damage to the esophageal muscle layer and reducing the risk of anastomotic leakage. Secondly, the application of SPLT can better expose the esophagus, and if necessary, intraoperative gastroscopy can be used. The combination of the two can obtain a higher cutting edge and ensure negative resection margins, which is more suitable for patients with Siewert type II and obesity. Ligation of the sterile rope can avoid gastric juice overflow after opening the esophagus, effectively block the spread of the tumor, and reduce the risk of abdominal infection. The linear stapler was placed from the side of the surgeon, which improved the anastomosis effect and reduced the anastomosis time. In addition, when performing ‘Y-loop’ anastomosis outside the abdominal cavity, we recommend reserving a 5 cm length for anastomosis at the distal jejunum stump. When using a 45 mm linear stapler for anastomosis, since the length of the stapler is shorter than the reserved intestinal tube, it can effectively reduce the tension of the anastomosis, thereby reducing the risk of jejunal perforation and anastomotic leakage. Different from the circular stapler with two rows of nails, we used a 45 mm linear stapler with three rows of nails to make the anastomosis wider and significantly reduce the incidence of anastomotic stenosis. Thirdly, the use of power shovel instruments can open the lower esophagus more smoothly, and at the same time, accurate and effective hemostasis can be achieved. After opening, a gastric tube was inserted as a guide to insert a linear stapler to ensure that no esophageal submucosal false passage was formed. After the anastomosis was completed, the gastric tube was inserted again to check whether the anastomosis was unobstructed, and the inflation test was performed to ensure that the common opening was completely closed. One of the key steps of overlap anastomosis is the closure of the common opening, which requires the surgeon to have a higher laparoscopic suture technique. In our experience, the surgeon changes the position, sutures the common opening on the assistant side, takes the anastomosis nail as the axis, takes the esophageal stump as the midpoint, and uses the barbed line from left to right to suture 6–8 needles continuously. After the suture is completed, the inflation experiment is carried out through the gastric tube to ensure the suture quality. Finally, we adopted the anastomosis behind the transverse colon, which significantly shortened the pull-up distance of the distal jejunum, thus effectively reducing the anastomotic tension and avoiding the problems of jejunum compression and abdominal wall adhesion in the anastomosis in front of the colon.

This study has some limitations. First, the sample size was relatively small. Although the data of 158 patients provide us with some statistical power, a larger sample size may be more conducive to discovering subtle differences between surgical methods. Secondly, the sample selection may have introduced selection bias, because all patients were selected from a single center, which may not fully represent all patients with gastric cancer. For example, if these patients differ from the general population in terms of the occurrence and progression of the disease or the socio-economic status, then our results may not be generalizable to the wider patient population. Although SPLT technology has shown good safety and efficacy in this study, whether these findings are applicable to a wider group of gastric cancer patients, or whether they are equally effective in a resource-limited clinical environment, remains to be further studied. Third, since the data were collected retrospectively, we relied on the accuracy and completeness of historical medical records. If the information was incomplete or wrong, our conclusions may be affected. This may lead to information bias. In addition, measurement bias may occur when evaluating postoperative complications, because there may be subjective judgments regarding the severity of complications, which may affect our assessment of technical safety.

These limitations may affect our interpretation of the efficacy and safety of SPLT technology; thus, we set clear inclusion and exclusion criteria to ensure the representativeness of the study population and to minimize the impact of selection bias on the results. In terms of data collection, we adopted a standardized data collection and measurement process to improve the quality of data. At the same time, regular training was carried out to ensure that all clinicians and data loggers involved in the study had a clear and consistent understanding of the data collection standards. We use a unified data extraction table to ensure that all researchers collected data according to the same criteria. Before the analysis, the data were rigorously screened to exclude incomplete or low-quality records, and the data were processed using a consistent standardized method. Through these measures, we aimed to reduce the information and measurement bias. We also asked multiple researchers to independently extract and analyze data, and then compared the results to reduce the impact of personal bias on the results.

Since the main purpose of this study was to evaluate the safety and efficacy of SPLT in the short term, we did not conduct long-term analyses. However, the preliminary results are encouraging. In the future, long-term follow-up will be essential to evaluate the clinical efficacy of the technique. In future studies, we recommend using a prospective design to more accurately control variables to minimize the impact of these biases, and to try to conduct research in multiple centers to increase sample diversity and research generalizability. Considering that regional differences may affect medical practice and patient characteristics, domestic and international multicenter studies are needed to verify the generalizability of SPLT technology.

## 5 Conclusion

We used SPLT to perform overlap anastomosis in TLTG. We only evaluated patients over the short-term; however, our preliminary results show that SPLT is a safe and reliable surgical method, which can simplify the procedure of digestive tract reconstruction, and ensure the safety of the cutting edge. In the future, we hope to design prospective studies to more objectively evaluate the clinical efficacy of this technique.

## Data availability statement

The raw data supporting the conclusions of this article will be made available by the authors, without undue reservation.

## Ethics statement

The studies involving humans were approved by Ethics Committee of the Second Affiliated Hospital of Fujian Medical University. The studies were conducted in accordance with the local legislation and institutional requirements. Written informed consent for participation was not required from the participants or the participants’ legal guardians/next of kin because This is a retrospective study. According to national legislation and institutional requirements, this study does not require written informed consent to participate.

## Author contributions

JW: Methodology, Project administration, Writing – original draft, Writing – review & editing. JX: Software, Writing – original draft. PW: Data curation, Writing – original draft. JL: Writing – original draft. WZ: Writing – original draft. WK: Writing – original draft. CW: Writing – original draft. JC: Writing – original draft. HZ: Writing – original draft. KY: Project administration, Supervision, Writing – original draft, Writing – review & editing.

## References

[1] Kitano S Iso Y Moriyama M Sugimachi K. Laparoscopy-assisted Billroth I gastrectomy [published correction appears. Surg Laparosc Endosc. (1994) 4:146–8. 8180768

[2] Etoh T Ohyama T Sakuramoto S Tsuji T Lee SW Yoshida K. Five-year survival outcomes of laparoscopy-assisted vs open distal gastrectomy for advanced gastric cancer: The JLSSG0901 randomized clinical trial. JAMA Surg. (2023) 158:445–54. doi: 10.1001/jamasurg.2023.0096 PMC10018406 36920382

[3] Aiolfi A Sozzi A Bonitta G Lombardo F Cavalli M Campanelli G. Short-term outcomes of different esophagojejunal anastomotic techniques during laparoscopic total gastrectomy: a network meta-analysis. Surg Endosc. (2023) 37:5777–90. doi: 10.1007/s00464-023-10231-6 37400689

[4] Sozzi A Aiolfi A Matsushima K Bonitta G Lombardo F Viti M. Linear-versus circular-stapled esophagojejunostomy during total gastrectomy: systematic review and meta-analysis. J Laparoendosc Adv Surg Tech A. (2023) 33:524–33. doi: 10.1089/lap.2023.0004 37057962

[5] Omori T Oyama T Mizutani S Tori M Nakajima K Akamatsu H. A simple and safe technique for esophagojejunostomy using the hemidouble stapling technique in laparoscopy-assisted total gastrectomy. Am J Surg. (2009) 197:e13–7. doi: 10.1016/j.amjsurg.2008.04.019 19101245

[6] Inaba K Satoh S Ishida Y Taniguchi K Isogaki J Kanaya S. Overlap method: novel intracorporeal esophagojejunostomy after laparoscopic total gastrectomy. J Am Coll Surg. (2010) 211:e25–9. doi: 10.1016/j.jamcollsurg.2010.09.005 21036074

[7] Hong J Wang YP Wang J Bei YB Hua LC Hao HK. A novel method of self-pulling and latter transected reconstruction in totally laparoscopic total gastrectomy: feasibility and short-term safety. Surg Endosc. (2017) 31:2968–76. doi: 10.1007/s00464-016-5314-y 27826782

[8] Wang FH Shen L Li J Zhou ZW Liang H Zhang XT. The Chinese Society of Clinical Oncology (CSCO): clinical guidelines for the diagnosis and treatment of gastric cancer. Cancer Commun (Lond). (2019) 39:10. doi: 10.1186/s40880-019-0349-9 30885279 PMC6423835

[9] Mou TY Hu YF Yu J Liu H Wang YN Li GX. Laparoscopic splenic hilum lymph node dissection for advanced proximal gastric cancer:a modified approach for pancreas-and spleen-preserving total gastrectomy. World J Gastroenterol. (2013) 19:4992–9. doi: 10.3748/wjg.v19.i30.4992 PMC3740431 23946606

[10] Japanese Gastric Cancer Association. Japanese gastric cancer treatment guidelines 2018 (5th edition). Gastric Cancer. (2021) 24:1–21. doi: 10.1007/s10120-020-01042-y 32060757 PMC7790804

[11] Dindo D Demartines N Clavien PA. Classification of surgical complications: a new proposal with evaluation in a cohort of 6336 patients and results of a survey. Ann Surg. (2004) 240:205–13. doi: 10.1097/01.sla.0000133083.54934.ae PMC1360123 15273542

[12] Facy O Arru L Azagra JS. Intestinal anastomosis after laparoscopic total gastrectomy. J Visc Surg. (2012) 149:e179–84. doi: 10.1016/j.jviscsurg.2012.04.009 22633089

[13] Du J Shuang J Li J Li J Hua J. Intracorporeal circular-stapled esophagojejunostomy after laparoscopic total gastrectomy: a novel self-pulling and holding purse-string suture technique. J Am Coll Surg. (2014) 218:e67–72. doi: 10.1016/j.jamcollsurg.2013.11.023 24559969

[14] Amisaki M Kihara K Endo K Suzuki K Nakamura S Sawata T. Comparison of single-stapling and hemi-double-stapling methods for intracorporeal esophagojejunostomy using a circular stapler after totally laparoscopic total gastrectomy. Surg Endosc. (2016) 30:2994–3000. doi: 10.1007/s00464-015-4588-9 26487216

[15] Kwon IG Son YG Ryu SW. Novel intracorporeal esophagojejunostomy using linear staplers during laparoscopic total gastrectomy: π-shaped esophagojejunostomy, 3-in-1 technique. J Am Coll Surg. (2016) 223:e25–9. doi: 10.1016/j.jamcollsurg.2016.06.011 27370184

[16] Edholm D. Systematic review and meta-analysis of circular-and linear-stapled gastro-jejunostomy in laparoscopic roux-en-y gastric bypass. Obes Surg. (2019) 29:1946–53. doi: 10.1007/s11695-019-03803-w 30864104

[17] Lee S Lee H Song JH Choi S Cho M Son T. Intracorporeal esophagojejunostomy using a linear stapler in laparoscopic total gastrectomy: comparison with circular stapling technique. BMC Surg. (2020) 20:100. doi: 10.1186/s12893-020-00746-3 32398072 PMC7218545

[18] Okabe H Obama K Tsunoda S Tanaka E Sakai Y. Advantage of completely laparoscopic gastrectomy with linear stapled reconstruction: a long-term follow-up study. Ann Surg. (2014) 259:109–16. doi: 10.1097/SLA.0b013e31828dfa5d 23549426

[19] Nagai E Ohuchida K Nakata K Miyasaka Y Maeyama R Toma H. Feasibility and safety of intracorporeal esophagojejunostomy after laparoscopic total gastrectomy: inverted T-shaped anastomosis using linear staplers. Surgery. (2013) 153:732–8. doi: 10.1016/j.surg.2012.10.012 23305598

[20] Huang ZN Huang CM Zheng CH Li P Xie JW Wang JB. Digestive tract reconstruction using isoperistaltic jejunum-later-cut overlap method after totally laparoscopic total gastrectomy for gastric cancer: short-term outcomes and impact on quality of life. World J Gastroenterol. (2017) 23:7129–38. doi: 10.3748/wjg.v23.i39.7129 PMC5656460 29093621

[21] Xinhua C Tian L Huilin H Mingli Z Tao C Hao C. Application value of overlap guiding tube(OGT) in assisting overlap esophagojejunostomy during laparoscopic total gastrectomy for gastric/gastroesophageal junction (G/GEJ) tumors. Gastric Cancer. (2022) 25:827–36. doi: 10.1007/s10120-022-01296-8 PMC9225966 35460378

[22] Kawamura H Ohno Y Ichikawa N Yoshida T Homma S Takahashi M. Anastomotic complications after laparoscopic total gastrectomy with esophagojejunostomy constructed by circular stapler (OrVil™) versus linear stapler (overlap method). Surg Endosc. (2017) 31:5175–82. doi: 10.1007/s00464-017-5584-z 28488177

[23] Hirahara N Matsubara T Hayashi H Takao S Hyakudomi R Yamamoto T. Overlapping esophagojejunostomy using a linear stapler in laparoscopic total or proximal gastrectomy. J Laparoendosc Adv Surg Tech A. (2023) 33:988–93. doi: 10.1089/lap.2023.0027 37172302

[24] Cui X Zhang S Du T Jiang X. Improvement of esophagojejunostomy technique after total gastrectomy with overlap method: reduce the difficulty of surgery and shorten operation time. Updates Surg. (2023) 75:1355–60. doi: 10.1007/s13304-023-01526-3 37166621

[25] Chen D Yang F Woraikat S Tang C Qian K. Effectiveness and safety of self-pulling and latter transected Roux-en-Y reconstruction in totally laparoscopic distal gastrectomy. Front Oncol. (2022) 12:916692. doi: 10.3389/fonc.2022.916692 36276133 PMC9585270

[26] Aslan M Topgül K. A novel, easier and safer alternative method for oesophagojejunal reconstruction after totally laparoscopic total gastrectomy. Surg Endosc. (2023) 37:4075–83. doi: 10.1007/s00464-023-09992-x 36952045

[27] Qiu XT Zheng CY Liang YL Zheng LZ Zu B Chen HH. Totally laparoscopic total gastrectomy using the "enjoyable space" approach coupled with self-pulling and latter transection reconstruction versus laparoscopic-assisted total gastrectomy for upper gastric cancer: short-term outcomes. Wideochir Inne Tech Maloinwazyjne. (2022) 17:352–64. doi: 10.5114/wiitm.2022.113568 PMC9186084 35707341

[28] Wan H Xiong J Chen Y Wei H Tang R Chen C. Application of half-transected and self-pulling esophagojejunostomy in total laparoscopic gastrectomy for gastric cancer: A safe and feasible technique. Can J Gastroenterol Hepatol. (2022) 2022:2422274. doi: 10.1155/2022/2422274 35734016 PMC9208976

